# Exploring the causes of learning burnout in online learning among master’s students: a Q-methodology study

**DOI:** 10.3389/fpsyg.2026.1704567

**Published:** 2026-03-23

**Authors:** Shunli Zuo, Yan Li, Jingling Li, Xiuyuan Zhu, Ning Liu, Ji Bo Wang

**Affiliations:** 1 Faculty of Nursing, Zhuhai Campus of Zunyi Medical University, Zhuhai, China; 2 Department of Science, The Fifth Affiliated Hospital of Zunyi Medical University (Zhuhai), Zhuhai, China; 3 Department of Rehabilitation Therapy, Zhuhai Campus, Zunyi Medical University, Zhuhai, China; 4 Department of Experimental Management at Academic Affairs Office, Zunyi Medical University, Zhuhai, Guangdong, China

**Keywords:** learning burnout, masters students, online learning, Q methodology, qualitative study

## Abstract

**Purpose:**

This study aims to identify the causes of learning burnout among master’s students participating in online learning through the Q methodology. The findings are intended to inform course design and instructional practices for graduate educators.

**Methods:**

This study utilized Q methodology to investigate the factors contributing to learning burnout among master’s students in online environments. The research followed these steps: (1) defining Q-channels, (2) constructing the Q-sample, (3) selecting the P-sample, (4) conducting Q-ranking, and (5) analyzing data and interpreting results.

**Results:**

Fifteen master’s students participated in the study. The identified causes of online learning burnout were categorized into four groups: (1) lack of self-management and academic pressure, (2) external interference and lack of motivation, (3) challenges in adapting to the online classroom environment, and (4) distractions and limited learning resources.

**Conclusion:**

The causes of online learning burnout among postgraduate students are complex and varied. To alleviate this burnout, targeted interventions are essential for improving the quality of online learning and for supporting the well-being and academic progress of master’s students.

## 1 Introduction

Postgraduate education plays a vital role in higher education, and the academic development of master’s students has garnered significant attention from scholars and educators (Greenhow et al., 2022). In recent years, the rapid advancement of information technology has made online learning an increasingly essential component of modern education. According to the theory proposed by Schaufeli et al. (2002), academic burnout is characterized by emotional exhaustion stemming from academic demands, a cynical and disengaged attitude toward academic tasks, and a diminished sense of competence in fulfilling academic obligations. Online learning transcends the temporal and spatial constraints of traditional education, allowing master’s students to establish more flexible study schedules and address personalized learning needs. However, survey data show (Farina et al., 2020; Nurani et al., 2022) that a substantial proportion of graduate students experience varying degrees of learning burnout during prolonged online instruction (e.g., nearly 40% in some cohorts). This burnout manifests as reduced interest in learning, diminished motivation, and depressive symptoms, among other effects. The persistence of academic burnout adversely affects students’ mental health and may even lead to severe outcomes such as suicide (Amelia and Science, 2022; Lu et al., 2023). This issue undermines not only the academic performance and psychological well-being of graduate students but also has the potential to negatively influence their future career development. Compared to traditional face-to-face lectures, online learning introduces significant differences in both the learning environment and modes of interaction.

Most research on the causes of learning burnout primarily adheres to classical motivational theories and extended models. However, there is a significant deficiency of systematic, context-specific bottom-up empirical studies that account for the cognitive, affective, and social developmental characteristics of Chinese master’s students, as well as the use of self-assessment methods to investigate the causes of burnout. This gap highlights the need for improved research design and methodologies for analyzing bottom-up empirical data. Q methodology, a scientific approach for examining human subjectivity, merges the strengths of both quantitative and qualitative research while emphasizing the subjectivity and individuality of participants (Churruca et al., 2021). Utilizing the Input-Environment-Output (IEO) model as the primary analytical framework, this study systematically integrates four foundational theories to construct a multi-dimensional explanatory system.

First, from the “input” perspective, self-determination theory (SDT) elucidates how the satisfaction of basic psychological needs—autonomy, competence, and relatedness—in online learning influences intrinsic motivation, a fundamental “input” factor. Second, concerning the “environment” dimension, conservation of resources (COR) theory delineates how the loss of critical resources (e.g., inadequate learning materials, limited social support) and an imbalance between resource gain and depletion in the online context contribute to burnout. Third, from the “output” perspective, self-worth theory indicates that students experiencing academic pressure in online settings tend to display self-protective avoidance behaviors, a significant “output” manifestation of burnout. Finally, the job demand-resource (JD-R) model complements this framework by highlighting the dynamic interplay between environmental demands (e.g., academic pressure, technical challenges) and personal resources (e.g., self-management skills, intrinsic motivation), thereby refining the analysis of the “environment-input” interaction in shaping burnout. Guided by this integrated theoretical framework, Q methodology is employed to identify and categorize the subjective determinants of online learning burnout among master’s students. The findings are anticipated to inform targeted interventions aimed at alleviating online learning burnout in Chinese master’s students and to enhance the theoretical integration of burnout research within online higher education contexts.

## 2 Literature review

Learning burnout poses a significant challenge in higher education, manifesting as emotional exhaustion, depersonalization, and a reduced sense of personal accomplishment (Maslach et al., 2001). Schaufeli et al. (2002) further define it as a psychological state that arises from prolonged academic stress, resulting in emotional exhaustion, alienation from learning, and decreased academic efficacy. To systematically investigate this phenomenon, this study employs the Input-Environment-Output (IEO) model (Astin, 1993), which has been adapted by Chinese scholars (Li, 2022) to suit local contexts, as its primary framework. This model asserts that learning burnout—a maladaptive psychological “output”—emerges from the dynamic interaction between individual “input” characteristics (e.g., motivation, personality) and situational “environmental” factors (e.g., institutional culture, teaching models). Within this framework, initial motivational resources are gradually depleted due to persistent environmental pressures, ultimately leading to burnout symptoms across emotional, cognitive, and behavioral dimensions.

The dimensions of the IEO model are elucidated through several complementary theories. From the “input” perspective, Self-Determination Theory (SDT) underscores the significance of satisfying fundamental psychological needs—autonomy, competence, and relatedness (Klein, 2019)—for sustaining motivation and well-being. Conversely, environments that obstruct these needs, as shown by Gong et al. (2023) correlate with heightened burnout. The “environment” dimension is further clarified by the Job Demands-Resources (JD-R) model and Conservation of Resources (COR) theory, which conceptualize burnout as arising from an imbalance between contextual demands (e.g., academic pressure, distractions) and available resources (e.g., social support, learning materials). Finally, regarding “output,” Self-Worth Theory interprets maladaptive coping behaviors (e.g., procrastination, avoidance) as strategies employed to protect self-esteem in competitive environments (Covington, 1992), which ultimately exacerbates feelings of alienation and burnout.

**FIGURE 1:**
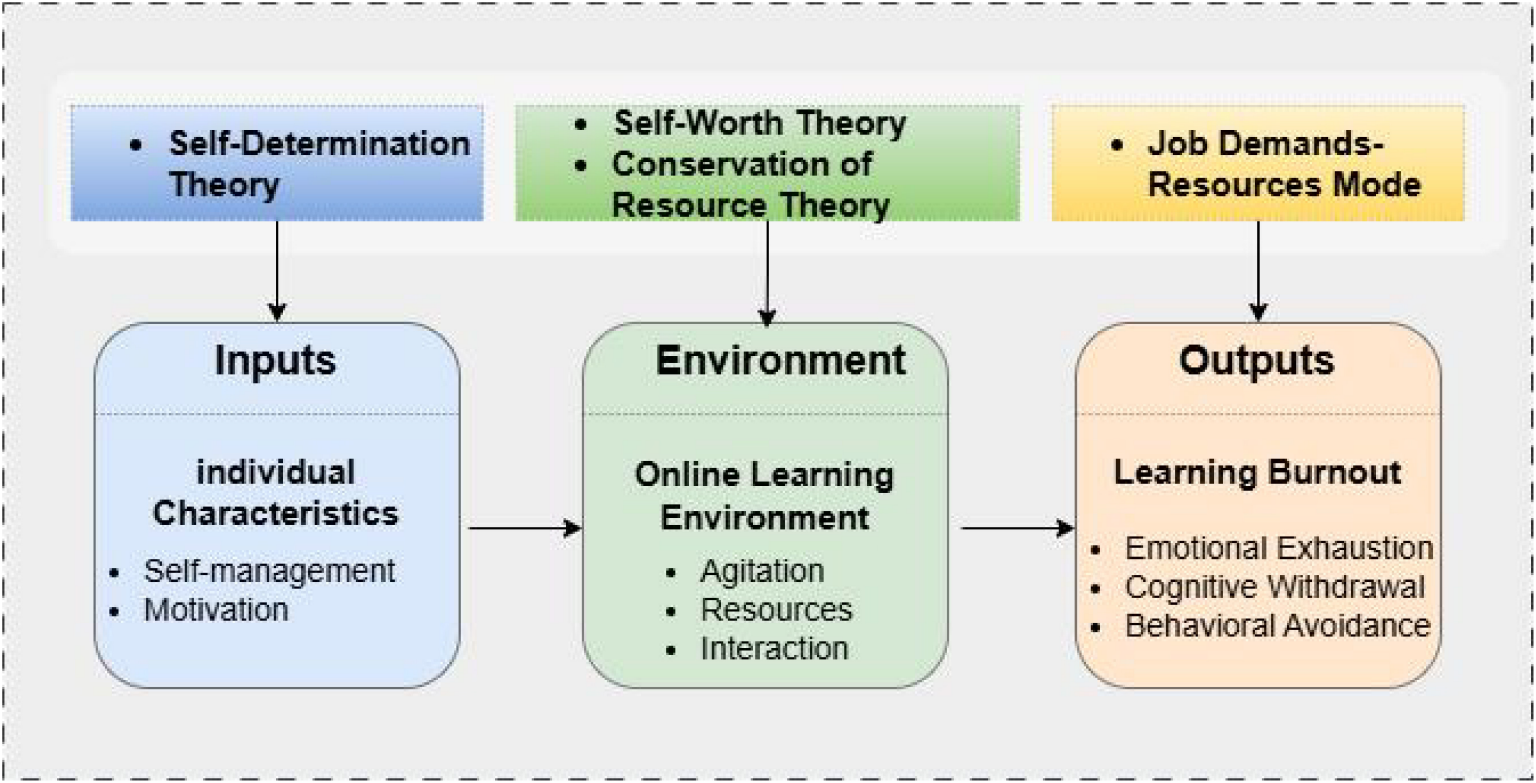
A comprehensive theoretical framework of online learning burnout based on the IEO model.

Contextualizing these mechanisms within specific cultural settings is crucial. Research conducted by Kossewska et al. (2023) and Tomaszek and Muchacka-Cymerman (2020, 2021, 2022) underscores the influence of factors such as coping strategies and emotional regulation across diverse educational systems. In the Chinese context, characterized by high academic expectations, collectivist norms, and exam-oriented structures, distinct pathways to burnout are likely to arise, necessitating a comprehensive, culturally informed investigation. However, existing research predominantly utilizes a “top-down” theoretical approach, neglecting methods that capture students’ subjective experiences from a “bottom-up” perspective. The Q method, which merges quantitative rigor with qualitative depth, effectively addresses this methodological gap by capturing students’ subjective perspectives through a “bottom-up” approach. Guided by the integrated theoretical framework of this study, the construction of the Q-sample (e.g., statements regarding motivation, resource availability, and self-protective behaviors) and the interpretation of factors are systematically aligned with the core propositions of self-determination theory, COR theory, self-worth theory, and the JD-R model. This integration ensures that the subjective viewpoints captured by Q methodology are not only descriptive but also theoretically explanatory. Consequently, this method is particularly well-suited for examining the subjective understanding and internal logical structure of burnout among Chinese postgraduate students, while elucidating the multi-layered mechanisms underlying online learning burnout. A visual representation of this integrated theoretical framework is presented in Figure 1.

## 3 Research methods

The Q method examines the structure of individual subjectivity through statistical data analysis (Stephenson, 1953) aligning well with the integrated theoretical framework of this study. This embedded hybrid research approach combines qualitative insights with quantitative measures, enabling the systematic mapping of participants’ subjective perspectives onto theoretical constructs such as motivation, resource loss, and self-protection. The implementation process, guided by the core theories, can be broadly categorized into five steps (Shen et al., 2020): defining Q-channels (based on theoretical dimensions such as motivation, environment resources, and self-worth), constructing the Q-sample (aligned with theoretical propositions to ensure coverage of key factors), selecting the P-sample (maximizing heterogeneity to capture diverse theoretical-relevant perspectives), conducting Q-ranking, and performing data analysis and result interpretation (linking factor types to theoretical mechanisms).

### 3.1 Q-concourse

The Q method is executed by developing a Q-concourse (statement library) to systematically gather and identify all potential viewpoints and opinions on a specific topic (Watts and Stenner, 2012). To thoroughly investigate the underlying causes of online learning burnout among postgraduate students, this study employed a qualitative research approach and established an initial statement database by integrating semi-structured interviews with literature analysis.

We conducted semi-structured interviews with 12 postgraduate students who possess extensive online learning experiences. Each interview lasted approximately 30–40 min and was held in a separate, quiet room, with the entire process recorded. Following the interviews, all audio recordings were transcribed verbatim for analysis. The interview outline (see Supplementary Appendix 1 for details) focused on 12 core questions designed to thoroughly explore the participants’ personal experiences and subjective feelings during the online learning process. By the 10th interview, data saturation was achieved, as no new core insights emerged. The 12 postgraduate students, comprising 7 females and 5 males, were aged between 24 and 33 and represented a range of disciplines and specialties to ensure diverse perspectives. We performed rigorous coding and thematic analysis on the transcribed text, and with the aid of NVivo 12.0 software, we ultimately identified five core dimensions from the interview materials: adaptation to the learning environment, maintenance of learning motivation, coping with academic pressure, quality of teaching interaction, and self-management effectiveness. Based on these dimensions, we generated 20 representative qualitative statements.

Theoretical statements are augmented through a systematic literature review. To strengthen the theoretical foundation of the statement database and expand the range of perspectives, this study conducted a comprehensive review of both domestic and international literature pertaining to online learning burnout. From the examination of influencing factors, formation mechanisms, and intervention strategies related to learning burnout, 55 representative theoretical statements were identified, addressing the potential limitations of a singular interview perspective. Ultimately, the 75 initial statements derived from interviews and literature in this study thoroughly encompassed all potential dimensions of online learning burnout among postgraduate students, thereby establishing a robust foundation for subsequent Q-sample screening and ranking.

### 3.2 Q-sample construction

A Q-sample comprises a collection of opinions on a specific topic, encompassing discrete ideas, concepts, or statements, ideally until saturation is achieved (Churruca et al., 2021). This process entails conducting a literature review, initial data collection—such as interviews—and exploration of public resources, followed by the simplification and refinement of the gathered statements to establish a Q-sequence set (Paige and Morin, 2016). In this study, the Q-sample was derived from interviews and a review of pertinent journal literature, supplemented by in-depth interviews. Two experts were invited to conduct repeated reviews and revisions. A total of 75 sentences were classified, organized, and streamlined, with sentences exhibiting semantic repetition eliminated. Ultimately, 32 sentences were selected (see Supplementary Appendix 2), resulting in a sample size of O equal to 32. Each Q-statement was then randomly numbered and printed on an 8.0 cm x 4.0 cm card, creating a stack of 32 Q-statement cards.

### 3.3 Select sample P

In Q methodology, the P-sample refers to the group of participants who sort the Q-statements. Rather than pursuing statistical representativeness, researchers intentionally assemble a small P-sample to encompass a variety of perspectives on the topic, thereby reflecting the spectrum of subjectivity pertinent to the issue. A fundamental selection principle is to maximize the heterogeneity of viewpoints instead of merely increasing the sample size. This approach allows the method to reveal distinct, shared perspectives within the concourse while utilizing resources efficiently. Consequently, guidelines generally advise that the P-sample should not exceed the number of Q-items. Yu and Chen (2010) suggest an upper limit calculated as N_*max*_ = (number of Q items/2) - 1.

In this study, the P-sample was selected based on the principle of maximum heterogeneity to ensure the representation of diverse perspectives on online learning burnout. Fifteen master’s students from a university in Zhuhai, Guangdong Province, all of whom had experienced online learning burnout, were purposively recruited to encompass key dimensions such as grade level (1st–3rd year), residency (urban vs. rural), and gender. This strategic variation aimed to reflect the range of experiences within the master’s student population. Such heterogeneity is essential in Q methodology, as it allows factor analysis to identify the principal distinct viewpoints present within the concourse, rather than emphasizing commonalities in a homogeneous group. The subsequent extraction of four robust and interpretable factors (see section 4.1) confirms that this sampling strategy effectively captured meaningful subjective diversity related to the research topic.

### 3.4 Q-ranking

**FIGURE 2:**
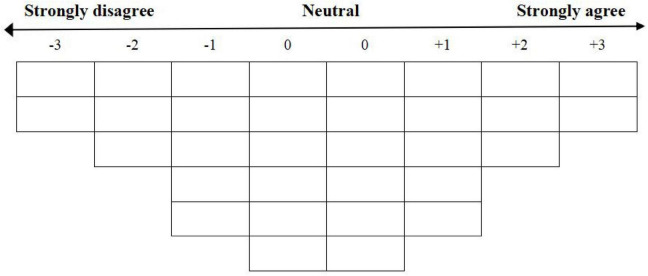
Q-sorting diagram (*N* = 32).

Based on the 32 items in the Q sample and relevant literature (Osborne et al., 2020), a 7-level normal distribution table was selected. This Q scale ranges from −3 to 3, where −3 indicates the least agreement, 3 signifies the most agreement, and 0 represents neutrality or uncertainty (for further details, refer to Figure 2). Additionally, it is essential that the sorter conducts the sorting process under the guidance of the researcher. The sorter should record the numbers of the two statements they most agree with in the far-right column (+3) of Supplementary Appendix 2, and the numbers of the two statements they least agree with in the far-left column (−3). Subsequently, the remaining statements should be filled in sequentially according to the degree of agreement. This process should be repeated until all statements are completed in the form without omissions or repetitions. Upon finishing the Q-sorting, the sorter must provide explanations for their Q statement rankings, particularly for the four statements designated as “most agree (+3)” and “least agree (−3),” which require detailed justification for the sorting decisions made.

### 3.5 Q analysis

In this study, we employed KADE (Ken-Q Analysis Desktop Edition) 1.3.1 software (Banasick, 2019) for data analysis. The Q-ranking results of the 15 P samples were systematically entered into the software using the provided data code, followed by subsequent analysis. (1) Factor analysis and rotation: Principal component analysis (PCA) was utilized to retain eight factors. We calculated the eigenvalues and variances (*R*^2^) for each factor, retaining those with eigenvalues exceeding 1. (2) Factor loadings: Factor loadings were computed to assess the strength of correlation between the samples and the factors, thereby elucidating the relationships among factors and aiding in their naming and interpretation (Akhtar-Danesh et al., 2008). (3) Factor interpretation: The structural characteristics of each factor were examined based on the *Z*-values of different P samples across the four factors. P samples with Z-values greater than +1.0 were classified as exhibiting positive views, while those with *Z*-values below −1.0 were classified as exhibiting negative views (Yeun, 2021).

## 4 Results

### 4.1 General information of the study participants

A total of 15 participants were included in this study, comprising 10 females (66.7%) and 5 males (33.3%). The ages of the participants ranged from 24 to 27 years. Specifically, 2 participants (13.4%) were in their 1st year of study, 8 (53.3%) were in their 2nd year, and 5 (33.3%) were in their 3rd year, as detailed in Table 1. Principal component analysis identified four factors, which were designated as follows: (1) self-management deficits and academic pressure type, (2) external interferences and lack of motivation type, (3) adaptation disorder in the online classroom environment, and (4) distraction and resource constraints type.

**TABLE 1:** Demographic characteristics of the four factors (*n* = 15).

Variables	*n*	Factor 1 (*n* = 6)	Factor 2 (*n* = 3)	Factor 3 (*n* = 3)	Factor 4 (*n* = 3)
Sex					
Male	5	1	1	1	2
Female	10	5	2	2	1
Age (years)					
23–24	4	1	0	1	2
25–26	6	4	1	1	0
≥ 27	5	1	2	1	1
Grade					
The first year of postgraduate study	2	1	0	0	1
Second year of postgraduate study	8	2	2	2	2
Third year of postgraduate study	5	3	1	1	0
Major					
Nursing science	2	0	0	1	1
Nursing	13	6	3	2	2
Residency					
Urban	7	3	1	0	3
Rural	8	3	2	3	0
Whether or not you are a class cadre					
Yes	6	2	1	1	2
No	9	4	2	2	1

The total number of valid P samples analyzed was 15, with the sample sizes allocated to each factor as follows: 6, 3, 3, 3, and 3. These correspond to explained variance ratios of 26, 19, 11, and 11%, respectively (for additional details, refer to Table 2).

**TABLE 2:** Eigenvalues and variance contributions of the four factors.

Items	Factor 1	Factor 2	Factor 3	Factor 4
Eigenvalue	3.86	2.78	1.72	1.59
Variance contribution rate (%)	26.00	19.00	11.00	11.00
Cumulative variance contribution rate (%)	26.00	45.00	56.00	67.00
Number of mapping samples (*n*)	6	3	3	3

### 4.2 Types of causes of online learning burnout among master’s degree students

#### 4.2.1 Factor 1—self-management deficit and academic stress

Type Six participants were assigned to Factor 1, comprising five females and one male. The corresponding participants were P1, P6, P9, P13, P14, and P15. Factor 1 represents burnout in online learning attributed to self-management deficits, such as inadequate planning and procrastination, alongside external academic stressors, including dissertation deadlines and multitasking demands. This factor emphasizes deficiencies in study planning (S15, S26), procrastination (S7), academic stress (S21, S22), and challenges related to multitasking (S23). Consequently, it is designated as the “self-management deficits and academic stress” type (for further details, please refer to Table 3).

**TABLE 3:** Self-management deficits and academic stress type-specific Q samples.

Q statement	Q sort	*Z*-scores
Q15. I have no study plan	3	2.04
Q21. I’ve been under a lot of pressure lately	3	1.73
Q26. I have no study goal	2	1.27
Q1. I feel the surroundings very hot	1	0.9
Q2. I saw someone else playing	1	0.6
Q27. When taking online classes, you can’t be as serious as in the classroom	0	0.02
Q28. The content of computer web pages attracts me	0	−0.03
Q8. I don’t think online courses are useful	−3	−2.18

A *z*> +1.0 is a positive view, and a *z*<−1.0 is a negative view.

#### 4.2.2 Factor 2—external interference and insufficient motivation

Type Three participants were assigned to Factor 2, comprising two females and one male, specifically P2, P8, and P12. This factor underscores burnout resulting from a combination of external distractions, such as electronic devices, and deficits in intrinsic motivation, exemplified by a tendency to give up when confronted with challenges. Key elements include distractions from electronic devices (S6), classroom interruptions (S5), reliance on external supervision (S24), and a lack of motivation (S12). Consequently, this factor is designated as the “external distractions and motivation deficits” type (Table 4).

**TABLE 4:** External disturbance and inadequate motivation type specificity Q sample.

Q statement	Q sort	*Z*-scores
Q5. I tend to be distracted in class	2	1.63
Q31. I often leave the online course and go to play it	−1	−1.09
Q28. The content of computer web pages attracts me	−2	−1.11
Q22. I have a lot of papers to write	−2	−1.32

A *z*> +1.0 is a positive view, and a *z*<−1.0 is a negative view.

#### 4.2.3 Factor 3—online class environment adaptation disorder type

Three participants, P4, P5, and P11, were assigned to Factor 3, which included two females and one male. This factor captures discomfort with the online teaching format, characterized by a perceived absence of classroom atmosphere and low content attractiveness, as well as a reliance on teacher supervision. It primarily highlights discomfort with the online class format (S27, S28), dependence on teacher oversight (S14), and unengaging content (S29). Consequently, this factor is designated as the “online class environment adaptation disorder” type (Table 5).

**TABLE 5:** Online classes environmental adaptation disorder type-specific Q sample.

Q statement	Q sort	*Z*-scores
Q27. When taking online classes, you can’t be as serious as in the classroom	2	1.4
Q14. I can’t study hard without my teacher	2	1.37
Q10. Network delay, I will not continue to study	−3	−1.8

A *z*> +1.0 is a positive view, and a *z*<−1.0 is a negative view.

#### 4.2.4 Factor 4—distraction and resource limitation type

Three participants, one female and two males, were assigned to Factor 4, specifically P3, P7, and P10. This factor highlights burnout resulting from restricted access to resources, such as paid courses, deficiencies in content design, and challenges in maintaining attention (Table 6).

**TABLE 6:** Attention distraction and resource-limited type-specific Q samples.

Q statement	Q sort	*Z*-scores
Q3. I see the desktop is messy	2	1.29
Q2. I saw someone else playing	0	−0.32
Q15. I have no study plan	−2	−1.38
Q14. I can’t study hard without my teacher	−3	−1.93
Q16. I don’t know what I need	−3	−2.35

A *z*> +1.0 is a positive view, and a *z*<−1.0 is a negative view.

## 5 Discussion

Guided by the integrated theoretical framework, this study identified four critical and interrelated dimensions that contribute to online learning burnout among master’s students: self-management ability, learning motivation, adaptation to the online environment, and resource limitations. These dimensions align with the core constructs of the IEO model and the supporting theories: (1) Self-management ability and learning motivation represent “input” factors elucidated by self-determination theory (e.g., competence and autonomy support) and self-worth theory (e.g., self-efficacy protection). (2) Adaptation to the online environment and resource limitations pertain to the “environment” dimension, which is central to COR theory (resource availability) and the JD-R model (environmental demands). (3) The four types of burnout (output) arise from the interplay of these input and environmental factors, with each type reflecting distinct theoretical mechanisms. These factors may co-occur and reinforce one another, thereby creating a cyclical process of burnout.

### 5.1 Self-management deficiency and academic pressure

Deficits in self-management and excessive academic pressure are primary contributors to online learning burnout. The JD-R model categorizes the former as “personal resources” and the latter as “environmental demands.” When demands consistently surpass resources, emotional exhaustion and cognitive disengagement are likely to occur. The self-worth theory further posits that students who lack self-discipline often resort to “procrastination” to safeguard their self-image, attributing potential failures to “lack of effort” rather than “incompetence,” thereby exacerbating burnout. All six interviewees highlighted “inability to make plans, task accumulation, and habitual procrastination,” which supports this mechanism. In a self-paced online learning environment, inadequate planning renders learning mechanical, gradually detaching emotions and ultimately leading to burnout. Previous studies have indicated that online courses characterized by weak self-regulation and minimal supervision pose the highest risk of burnout (Shao et al., 2022). For postgraduate students, the dual responsibilities of coursework and research further intensify time management pressures. Additionally, the “flexibility” inherent in online learning can lead to an underestimation of task difficulty, effectively doubling the perceived workload (Broadbent and Poon, 2015; Kahu and Nelson, 2018). Consequently, educators should integrate “teaching students self-regulation, goal setting, and time management” into their instructional design to bolster students’ resilience to stress and promote sustained engagement.

### 5.2 Environmental distractions and declining motivation

The second factor elucidates the combined effects of “environmental interference” and “decline of intrinsic motivation,” while simultaneously affirming both self-determination theory and conservation of resources theory. Self-determination theory posits that intrinsic motivation is sustained when the three fundamental needs of autonomy, competence, and relatedness are satisfied. However, social media interference in online contexts obstructs the fulfillment of these needs, thereby diminishing internal motivation for learning. Conservation of resources theory further conceptualizes digital interference as a “resource funnel,” wherein cognitive resources are continuously redirected, resulting in resource depletion and emotional exhaustion. All three interviewees exhibited a similar trajectory characterized by reliance on external supervision, avoidance of challenges, and addiction to social media and entertainment, reflecting the behavioral manifestations of these mechanisms. While digital tools facilitate flexible learning, they simultaneously serve as sources of distraction. Jose et al. (2015) found that frequent digital interruptions significantly impair sustained attention and cognitive performance. When intrinsic motivation wanes, students are likely to withdraw quickly when faced with challenging problems, leading to increased emotional exhaustion and alienation (Gong et al., 2023; Salmela-Aro and Upadyaya, 2014). This observation aligns closely with the central tenet of self-determination theory, which asserts that “the lack of autonomy and sense of competence weakens the internal driving force” (Ryan and Deci, 2000). Therefore, instructional design must incorporate a motivation support system that includes timely feedback, recognition mechanisms, and task meaningfulness to rekindle intrinsic motivation and maintain in-depth engagement.

### 5.3 Adaptation barriers in the online learning environment

The third dimension addresses the adaptation barriers arising from the characteristics of online education. The JD-R model characterizes this as the “absence of environmental resources,” where a lack of real-time interaction and inadequate teacher supervision result in a sudden increase in learning demands. Self-determination theory indicates that insufficient teacher-student interaction directly obstructs the “relatedness need,” leading to feelings of alienation and low engagement. Students frequently describe online learning as “disconnected, boring, and unmotivating,” aligning with theoretical expectations. Prior research has demonstrated that asynchronous classes exhibit significantly lower participation levels compared to synchronous classes, primarily due to the absence of social presence and immediate feedback (Hung et al., 2010; Richardson et al., 2017). Furthermore, the lack of non-verbal cues diminishes the sense of belonging and task value (Eccles and Wigfield, 2002). Expectancy-value theory posits that task value and situational quality collectively influence engagement levels. Consequently, universities must cultivate an online ecosystem that is “interaction-rich and socially fulfilling.” This requires a blend of collaborative projects, interactive forums, and multimedia teaching to enhance the community atmosphere and encourage sustained participation.

### 5.4 Resource limitations and cognitive overload

The fourth factor points directly to the triple threat of “resource scarcity—design flaws—environmental interference,” and its logic is highly isomorphic in the Conservation of Resources (COR) theory and the Job Demands—Resources (JD—R) model. The former views the absence of key resources such as high—quality content and quiet spaces as “continuous loss,” while the latter classifies poor content design as “a surge in learning demands” and insufficient resources as “a sudden drop in coping ability.” The combination of the two triggers cognitive overload and fatigue. The interviewees repeatedly mentioned “cluttered items, dim lighting, and boring content,” which is a concrete portrayal of the loss—imbalance mechanism. Yang and Jeon (2023) confirmed that a well-lit and ergonomic learning environment can significantly enhance attention persistence. Moreover, the economic pressure brought by paid courses further squeezes the psychological margin and dilutes the sense of learning value (Oana and Mitrea, 2021), which is in line with the core proposition of COR that “burnout occurs when resources are depleted” (Hobfoll, 1989). Therefore, institutions must simultaneously promote “resource equity” and “design optimization”: opening up high—quality digital textbooks, improving physical learning conditions, and streamlining cognitive load. Only by enabling students from different economic backgrounds to “have access to resources and reduce demands” can the source of burnout be blocked.

### 5.5 Interconnectedness and dynamic interaction among factors

This study importantly demonstrates that the four dimensions—self-management, motivation, adaptation, and resource limitations—are interconnected and mutually reinforcing. For example, inadequate self-management may result in a decline in motivation, which subsequently hinders adaptation to autonomous online learning environments. Likewise, insufficient adaptation and resource barriers, such as poor content design or digital fatigue, can further diminish motivation and self-discipline, thereby creating a cyclical mechanism of burnout. This interpretation aligns with the meta-analysis by Zhao et al. (2025), which conceptualized academic burnout as a multidimensional and dynamic construct influenced by the interplay of emotional exhaustion, resource imbalance, and contextual stressors. Consequently, burnout should be regarded not as a static psychological outcome but as a recursive process shaped by behavioral, emotional, and environmental factors.

### 5.6 Practical and theoretical implications

The findings of this study have significant practical implications for teaching and contribute theoretically to burnout research. First, universities should implement structured training focused on self-regulation, goal management, and time planning to enhance students’ autonomy and resilience. Second, educators can integrate motivation-support mechanisms, such as timely feedback, progress tracking, and gamification elements, to sustain student engagement. Third, AI-based adaptive learning systems may be utilized to monitor learning behaviors and emotional states, facilitating the early identification of burnout risk. Fourth, policymakers and institutions should prioritize the reduction of environmental and resource inequities by providing accessible, high-quality online platforms and free digital content. Theoretically, this study enriches the academic burnout literature by integrating the JD-R (Job Demands–Resources) and COR frameworks with Q methodology, demonstrating how cognitive, motivational, and contextual stressors interact to produce burnout among Chinese graduate students. Furthermore, it highlights the cultural particularities of burnout in high-pressure academic environments, where collectivist norms and achievement-oriented expectations exacerbate students’ stress responses.

## 6 Research limitation and future research directions

This study utilized Q methodology to investigate the causes of online learning burnout among master’s students; however, several limitations warrant acknowledgment. First, the sample size is relatively small and geographically concentrated, with participants primarily from medical and nursing disciplines, which limits the representativeness and generalizability of the findings. Second, the subjective nature of Q-sorting and factor interpretation may introduce researcher bias. Third, the cross-sectional design restricts the examination of how burnout evolves over time. Furthermore, the study did not consider potential moderating or mediating factors—such as coping styles, personality traits, institutional support, or technological proficiency—that may affect burnout trajectories. Future research should broaden the sample scope, include participants from diverse academic fields and multiple institutions, employ longitudinal designs to capture dynamic changes, and integrate a wider array of psychological, social, and contextual variables to foster a more comprehensive understanding of online learning burnout.

## 7 Conclusion

This study enhances the existing literature by identifying four interrelated factors that contribute to online learning burnout among master’s students and by offering a theoretically informed, practically applicable framework for intervention. By addressing self-management, motivation, environmental adaptation, and resource limitations—both individually and collectively—educators and institutions can foster a more supportive and sustainable online learning ecosystem that promotes student well-being and academic success.

## Data Availability

The original contributions presented in the study are included in the article/Supplementary material, further inquiries can be directed to the corresponding authors.
