# Supplementary material for: Exploring the causes of learning burnout in online learning among master’s students: a Q-methodology study
Source: Front Psychol. 2026 Mar 23;17:1704567. doi: 10.3389/fpsyg.2026.1704567 (PMC13050881; doi:10.3389/fpsyg.2026.1704567)
Supplement: Supplementary file 1 [file Supplementary_file_1.docx]

**Appendix 1**

**Interview outline**

Hello! First of all, thank you for your participation. I would like to interview you about the issue of learning burnout in online learning, so as to provide a reference for our research on the causes and mechanisms of online burnout learning. This interview is anonymous. The content of the answers is only for scientific research. Please feel free to answer.

1. Grade level and academic major;
2. What prior online learning experiences have you had?
3. What activities do you typically engage in after logging into the online learning platform?
4. Do you design a study plan before the course starts?
5. What strategies do you think can help you complete study tasks more efficiently?
6. Do you find the current design of learning activities in the online course reasonable?
7. What role do you think the instructor plays in online courses?
8. What challenges have you faced during the online learning process?
9. What were your expectations before the course began?
10. What aspects of the online learning experience were most satisfactory and unsatisfactory?
11. Have you ever considered dropping the online course, and if so, why?
12. What are your views on the future development of online learning?

This concludes the interview. Thank you for your cooperation and participation!
